# High-Efficiency Metamaterial-Engineered Grating Couplers for Silicon Nitride Photonics

**DOI:** 10.3390/nano14070581

**Published:** 2024-03-27

**Authors:** William Fraser, Radovan Korček, Ivan Glesk, Jan Litvik, Jens H. Schmid, Pavel Cheben, Winnie N. Ye, Daniel Benedikovic

**Affiliations:** 1 Silicon Micro/NanoPhotonics Group, Carleton University, Ottawa, ON K1S 5B6, Canada; williamfraser3@cmail.carleton.ca (W.F.); winnie.ye@carleton.ca (W.N.Y.); 2 National Research Council Canada, Ottawa, ON K1A 0R6, Canada; jens.schmid@nrc-cnrc.gc.ca (J.H.S.); pavel.cheben@nrc-cnrc.gc.ca (P.C.); 3 Department Multimedia and Information-Communication Technology, University of Zilina, 010 26 Žilina, Slovakia; radovan.korcek@uniza.sk (R.K.); jan.litvik@uniza.sk (J.L.); daniel.benedikovic@uniza.sk (D.B.)

**Keywords:** integrated photonics, silicon nitride, surface grating couplers, subwavelength grating metamaterials, amorphous silicon, fiber–chip connections

## Abstract

Silicon nitride (Si_3_N_4_) is an ideal candidate for the development of low-loss photonic integrated circuits. However, efficient light coupling between standard optical fibers and Si_3_N_4_ chips remains a significant challenge. For vertical grating couplers, the lower index contrast yields a weak grating strength, which translates to long diffractive structures, limiting the coupling performance. In response to the rise of hybrid photonic platforms, the adoption of multi-layer grating arrangements has emerged as a promising strategy to enhance the performance of Si_3_N_4_ couplers. In this work, we present the design of high-efficiency surface grating couplers for the Si_3_N_4_ platform with an amorphous silicon (α-Si) overlay. The surface grating, fully formed in an α-Si waveguide layer, utilizes subwavelength grating (SWG)-engineered metamaterials, enabling simple realization through single-step patterning. This not only provides an extra degree of freedom for controlling the fiber–chip coupling but also facilitates portability to existing foundry fabrication processes. Using rigorous three-dimensional (3D) finite-difference time-domain (FDTD) simulations, a metamaterial-engineered grating coupler is designed with a coupling efficiency of −1.7 dB at an operating wavelength of 1.31 µm, with a 1 dB bandwidth of 31 nm. Our proposed design presents a novel approach to developing high-efficiency fiber–chip interfaces for the silicon nitride integration platform for a wide range of applications, including datacom and quantum photonics.

## 1. Introduction

Photonic integrated circuits (PICs) have become key components for a broad range of applications, spanning from telecommunications to high-performance optical and quantum computing [1,2,3]. By capitalizing on mature silicon complementary metal–oxide–semiconductor (CMOS) technology, PICs offer a valuable means to develop densely integrated optical systems with increasing complexity and enhanced functionality. Recently, hybrid photonic platforms that combine different optical materials on a single chip have shown the potential to outperform single-layer waveguide architectures [4,5,6]. Hybrid PICs can be formed either by heterogenous or monolithic integration. Various materials, waveguide platforms, or even multiple photonic chips can be connected with bonding techniques [7], direct growth methods [8], transfer printing [9], or optical fibers [10].

Large-scale photonic systems require efficient interconnections between optical devices. Despite numerous advances, their performance is typically limited by geometrical, material, and modal mismatches. Edge couplers and surface grating couplers are typically used for high-performance optical input/output (I/O) interfaces, connecting photonic chips to the external world [11]. Edge couplers offer low-loss and polarization-insensitive coupling over a wide wavelength band [12,13]. However, their accessibility is limited to the chip’s edge, restricting the design flexibility of the PIC. Edge couplers are often connected with lensed or high-numerical-aperture fibers [14]. Yet, when interfacing with standard single-mode fibers (SMF-28 [15]), complex designs and additional fabrication steps [12,13] are necessary. Conversely, grating couplers are compact and have no routing restrictions. This enables fast photonic die testing in large volumes without the need for transitional packaging or additional post-processing steps, making them suitable for high-density optical interconnects [16]. The ability to position I/O ports at arbitrary locations on the chip surface also enables the realization of multi-port free-space and fiber–chip interfaces. Furthermore, surface gratings can support circuit integration via optical interposers [17] and are widely used in beam forming and steering applications such as free-space optical communications or light detection and ranging systems (LiDAR) [18].

Single-layer surface grating couplers have been successfully demonstrated on different platforms [11,19], notably on silicon-on-insulator (SOI) and silicon nitride (Si_3_N_4_). The SOI platform is now established as a mature waveguide architecture for deploying a diverse library of integrated photonic devices with optical and optoelectronic performances while maintaining low-cost production in open-access foundries. However, SOI waveguides still suffer from several drawbacks. These primarily include a high sensitivity to fabrication errors, scattering losses due to sidewall roughness, and intrinsic optical loss due to free-carrier and two-photon absorption [20,21]. The Si_3_N_4_ photonic integration platform is a promising alternative to SOI that alleviates some of these issues. The wide transparency window ranging from the visible to mid-infrared spectral regions, ultra-low propagation losses, and improved sensitivity to fabrication errors are some of the attractive properties of Si_3_N_4_. Furthermore, mature high- or low-temperature deposition techniques such as low-pressure chemical vapor deposition (LPCVD) and plasma-enhanced chemical vapor deposition (PECVD) provide an additional degree of freedom for controlling the structural and optical properties of Si_3_N_4_ waveguides [22,23]. In particular, the film thickness, refractive index, hydrogen/silicon ratio, modal confinement, birefringence, and dispersion can all be tuned through the deposition process. In terms of optical coupling, both platforms provide moderate-to-high refractive index contrast, which yields strong optical confinement. However, this leads to a high modal mismatch between the on-chip waveguide and optical fiber modes.

To date, SOI and Si_3_N_4_ photonic integrated circuits are now capable of achieving close to 1 dB efficiency through careful optimization of the directionality and field overlap between the radiated beam and the optical fiber mode. The fiber-grating field overlap, typically limited to 80% for uniform structures [24], can be enhanced through near-field apodization [25,26,27] or the beam-focalizing technique [28,29]. Near-field apodization is generally implemented by varying the geometry of the grating coupler in order to control the coupling length, i.e., to regulate the amount of waveguide power that is radiated by each grating period. This can be accomplished by utilizing duty cycle optimization, subwavelength grating (SWG) refractive index engineering, or by employing multiple shallow etch steps [30]. With the beam focalization approach, the radiated grating beam is focused through free space to the target mode size onto the optical fiber, which is situated at a specific distance away from the surface of the chip. In this configuration, a longer grating structure can be used to diffract all of the power off-chip since the target Gaussian profile is produced at the focal point rather than the near-field. Therefore, the beam focalization technique is particularly effective for waveguide platforms with a smaller refractive index contrast such as Si_3_N_4_. However, while the near-field grating apodization does not substantially impact the grating coupler’s bandwidth, the beam focalization is intrinsically wavelength-dependent. Consequently, it introduces chromatic aberrations that reduce the optical bandwidth of the coupler.

Thicker waveguide cores are necessary to improve the directionality of single-layer waveguide couplers [31,32]. These thicker cores are typically supported with a custom thickness of buried oxide (BOX) to produce constructive interference upwards towards the fiber rather than down into the substrate. However, commercial wafers provide only a discrete set of waveguide and BOX thicknesses. Both are typically fixed by PIC foundries [33,34] for multi-project wafer (MPW) fabrication runs. To achieve near-unity directionality, grating couplers with backside-engineered substrates have been used [35,36,37]. These include selective removal of the Si substrate followed by chip metallization [36] or by the formation of double [37,38] and/or multiple [39] Bragg reflectors underneath the grating. But these require fabrication steps that are incompatible with standard foundry prototyping services [31,33]. Additionally, multiple etch steps have been used in single-layer devices to break the vertical grating symmetry and promote high directionality [40,41,42,43]. Grating couplers with interleaved trenches or *L*-shaped waveguide geometry have also been reported [43]. The etch depth control and inter-layer mask misalignment are the practical aspects that hinder the widespread utilization of these techniques.

Recently, hybrid grating couplers with multi-layer configurations have emerged as promising candidates for efficient optical coupling, leveraging advanced fabrication processes applied to the chip’s frontside [44,45,46,47,48,49,50,51,52,53,54,55,56]. Compared to a rather complex backside chip processing, implementing new material layers on the chip’s frontside is advantageous as it provides an additional degree of freedom to improve the photonic chip functionalities and can be more readily integrated into a standard chip-scale fabrication process. More specifically, such grating coupler designs utilize stacks of the same [44,45,46] or different [47,48,49,50,51,52,53,54,55,56] materials to enhance the coupling efficiency. Moreover, this also provides more opportunities for fiber–chip coupling, particularly in terms of multiband [55] or polarization-insensitive [56] operation. Near-unity grating directionality has been reported using dual-layer silicon and Si_3_N_4_ designs [44,45], poly-Si [47]- or amorphous Si [48]-assisted structures, or hybrid Germanium-on-SOI (Ge-on-SOI) [49], Si_3_N_4_-on-SOI [50,52], or Si-on-Si_3_N_4_ [53,54] waveguide couplers. However, such implementations require direct epitaxial growth within small grating trenches, precise layer-to-layer alignment control, or dedicated layer stacks combined with multiple etching steps. All of these processing steps can incur high costs and are highly sensitive to fabrication imperfections.

In this work, we present a novel grating coupler design that utilizes a hybrid α-Si/Si_3_N_4_ photonic platform with a single-etch fabrication process. The surface grating is implemented in an α-Si overlayer to take advantage of the higher refractive index contrast (Δ*n* ≈ 2.5) compared to Si_3_N_4_ (Δ*n* ≈ 1), considering an air-cladded device configuration. An inter-layer coupler comprised of a two-stage α-Si taper was designed to transfer the optical power between the amorphous silicon and silicon nitride layers through the evanescent tail of the waveguide mode. The performance of uniform gratings with this hybrid architecture was investigated to optimize the vertical dimensions of the grating as well as establish a baseline for this platform. The coupling efficiency was then further enhanced through subwavelength grating (SWG) metamaterial apodization. This approach achieves efficient optical coupling between the SMF-28 fiber and Si_3_N_4_ photonic chips, with simple integration required by standard foundry-level technology, while maintaining compatibility with CMOS frontend processing.

## 2. Design and Methodology

### 2.1. Operating Principle and Performance

Surface gratings couple light between optical fibers and integrated waveguides by diffraction. In a uniform surface grating coupler, each period of the grating is identical, possessing the same geometrical parameters, and thus diffraction characteristics. A coupler of this type is commonly referred to as uniform or non-apodized since the grating strength is constant along the device. In this case, the radiation is governed by the momentum conservation condition [24]:

(1) $$n_{c}\sin(\Theta_{k})=n_{fb}+\frac{k\lambda }{\Lambda }$$

where *n_c_* is the refractive index of the cladding material, *Θ_k_* is the fiber angle with respect to the surface normal, *k* is an integer denoting the diffraction order, *n_fb_* is the effective index of the Floquet–Bloch optical mode supported by the grating, and Λ and Λ are the operating wavelength and grating period along the propagation direction, respectively. It is worth noting that only the radiation orders for which sin(*Θ_k_*) is real will diffract the optical power out of the planar waveguide [24]. Typically, the period of the surface grating is designed to support first-order diffraction *(k* = −1) and a single emission angle.

The fraction of optical power that is coupled between the on-chip waveguide mode and the near-Gaussian optical fiber mode depends on several factors. The overall coupling efficiency can be calculated as follows:

*η* = (1 − *R* − *T*) × *D* × *OL* (2)

where *R* and *T* are the amounts of power reflected back from the grating towards the input waveguide or remaining at the end of the grating, respectively; *D* is the directionality of the grating, which is defined as the ratio between the upward radiation towards the fiber and the total power diffracted by the grating; and *OL* is the field overlap integral between the radiated beam and the optical fiber mode. The field overlap is calculated using the complex electric field to consider both the amplitude and phase distributions according to Equation (3).

(3) $$OL=\frac{\int |E_{G}E_{F}*{|}^{2}dA}{\int E_{G}E_{G}*dA\int E_{F}E_{F}*dA}$$

where *E_G_* is the complex electric field radiated by the grating coupler and *E_F_* is the complex electric field distribution of the optical fiber mode. The symbol “*” denotes the complex conjugate.

### 2.2. Material Platform Description

**Figure 1:**
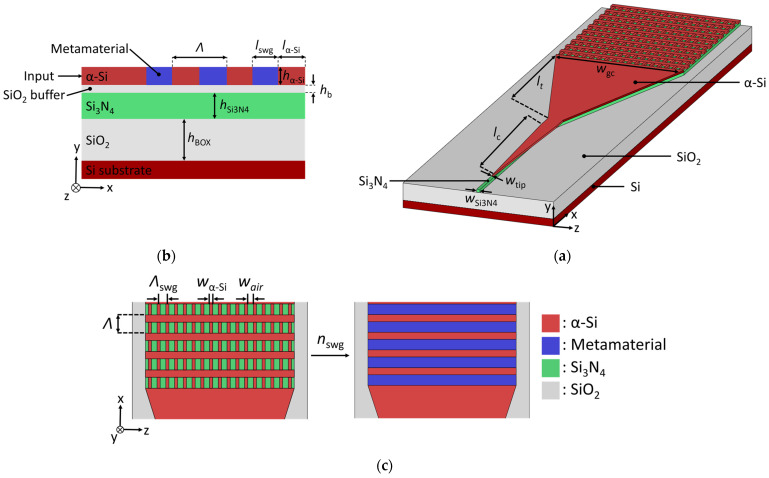
(**a**) Three-dimensional schematics of the SWG-engineered α-Si/Si_3_N_4_ grating coupler. (**b**) Corresponding side view (*x-y* plane) with structural grating parameters and (**c**) top view (*x*-*z* plane) of the grating coupler with SWG trenches and the corresponding coupler with synthesized equivalent index.

Our proposed grating coupler, schematically shown in Figure 1a, is implemented on a hybrid waveguide configuration comprising a Si_3_N_4_ wafer and an amorphous silicon overlay. The Si_3_N_4_ platform consists of a 400 nm thick waveguide core and 4.5 μm thick buried oxide (BOX) layer on a Si substrate. The α-Si surface grating of thickness *h*_α-Si_ and Si_3_N_4_ waveguide are separated by a silicon dioxide (SiO_2_) buffer of thickness *h*_b_. The cover medium is air. The use of high-index overlays has been demonstrated to improve the performance of a variety of passive and active integrated photonic devices [53,54,57,58]. For surface gratings, the deposition of high-index materials such as crystalline silicon (c-Si) [59], poly-Si [47], α-Si [54], or Ge [49] has been reported. In particular, α-Si has been used to break the vertical grating symmetry [48], leverage back-end-of-line CMOS metal layers for efficient and polarization-insensitive couplers [60], or improve the intrinsically low strength of Si_3_N_4_ grating couplers [16,51,53,54]. Here, the grating is entirely formed in the high-index layer, simultaneously increasing the grating directionality and strength, while maintaining compatibility with CMOS processes and single-etch fabrication. Moreover, the grating design leverages SWG metamaterials [61], as depicted in Figure 1c, to enhance the overlap between the near-Gaussian profile of the optical fiber mode and the radiated grating field. Our coupler is optimized for the transverse-electric (TE) waveguide mode and a central wavelength of 1.31 μm. This spectral range benefits from the low-dispersion characteristics of standard optical fibers, making it well suited for applications in short-reach datacom interconnects and quantum photonic applications, such as high-speed transceivers and quantum-dot heterogeneous integrated circuits [4]. At the nominal wavelength, the material indices of corresponding materials are *n*_α-Si_ = 3.5187, *n*_Si3N4_ = 2.0017, *n*_b_ = 1.4502, *n*_BOX_ = 1.4460, and *n*_air_ = 1. The coupler is designed using rigorous two-dimensional (2D) and three-dimensional (3D) finite-difference time-domain (FDTD) simulations with the Ansys Lumerical suite [62]. The design methodology relies on decoupled 2D simulations in the vertical (*x-y*) and the horizontal (*z-x*) planes to design the surface gratings and synthesize the SWG geometry, respectively. These 2D simulations can be decoupled since the grating width is much larger than the thickness of the waveguide layer (*w*_gc_ >> *h*_α-Si_) and reduces the computational time requirement. The final designs are then verified via rigorous 3D FDTD simulations.

### 2.3. SWG Metamaterials

**Figure 2:**
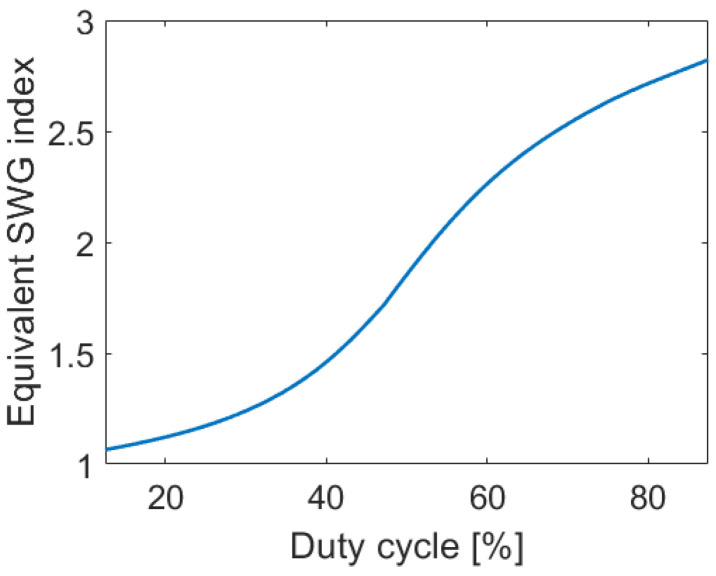
Synthesized equivalent refractive index as a function of SWG duty cycle, considering an SWG period of 400 nm.

The α-Si grating coupler uses SWG metamaterial structures formed in the grating trenches to control the grating strength, as depicted in Figure 1c. Since the first demonstration of SWG metamaterials in Si waveguides, they have become an essential photonic design tool, yielding superior device performances [61,63,64]. SWG metamaterials enable the manipulation of light propagation by engineering the refractive index [26], dispersion [65], modal confinement [66], and anisotropy [67] of the medium. In our design, the device acts as a classical diffraction-based grating along the direction of light propagation, while the SWG structure is patterned along the transverse direction (Figure 1c, *z*-axis), interleaving the etched trenches and non-etched solid blocks of amorphous silicon. Since the feature dimensions are smaller than the operating wavelength, losses and wavelength resonances due to reflection and diffraction effects are suppressed. As a result, the propagating field interacts with the SWG as if it were a homogenous medium with a refractive index dependent on the geometry and constituent materials [61,64]. An equivalent refractive index is synthesized by varying the width of the unetched (*w*_α-Si_) and etched (*w*_air_) blocks, i.e., by optimizing the lateral duty cycle, which is defined as the ratio of the unetched segment and the SWG period. The SWG period is chosen as Λ_swg_ < Λ_Bragg_ = Λ/2*n*_fb_, to ensure that a photonic bandgap does not open at the central wavelength. Here, Λ_Bragg_ is the first-order Bragg period, Λ is the operating wavelength (Λ = 1.31 µm, in free space), and *n*_fb_ is the effective index of the fundamental TE-polarized Floquet–Bloch mode in the grating region. Figure 2 shows the synthesized SWG metamaterials as a function of the SWG geometry. An SWG period of 400 nm was used for these calculations. The refractive index of the synthetic SWG metamaterial is calculated using a 2D multi-layer slab waveguide model in the *x-z* plane [26]. The final geometry of the SWG-engineered surface grating coupler, for both uniform and apodized designs discussed below, is then fine-tuned by rigorous 3D FDTD simulations.

### 2.4. Connecting Waveguides and Inter-Layer Coupler

**Figure 3:**
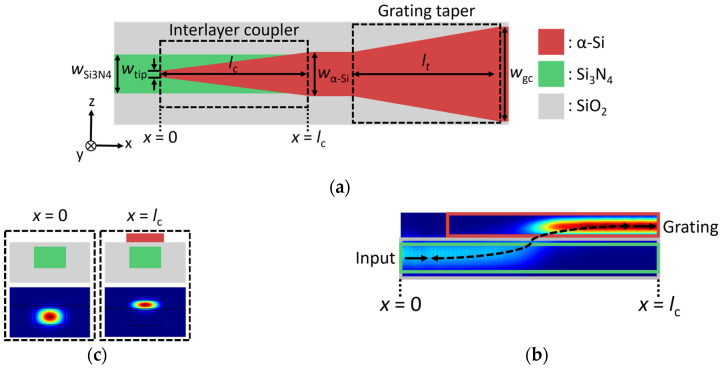
(**a**) Top-view (*x*-*z* plane) schematic of the inter-layer coupler. (**b**) Longitudinal view (*x-y* plane) of the optical intensity distribution in the inter-layer coupler obtained from 3D FDTD simulation. The fundamental TE mode is injected from the Si_3_N_4_ waveguide on the left-hand side. (**c**) Waveguide cross-sections (*y-z* plane) and corresponding field distributions of the quasi-TE fundamental modes for the single-mode Si_3_N_4_ and α-Si strip waveguides.

As illustrated in Figure 1, the grating coupler is implemented in the α-Si layer with light injection on the same plane. An inter-layer coupler is employed to transfer the light from the Si_3_N_4_ into an α-Si waveguide, as depicted in Figure 3a,b. In our hybrid platform, the thicknesses of the Si_3_N_4_, α-Si, and oxide buffer layers are fixed to 400 nm, 220 nm, and 50 nm. The Si_3_N_4_ thickness is given by the foundry [34], while the thicknesses of the buffer and the α-Si layers are optimized with respect to the grating coupler, as discussed in Section 3. The single-mode Si_3_N_4_ waveguide has a width (*w*_Si3N4_) of 850 nm. The input light from the Si_3_N_4_ waveguide is transferred into the upper α-Si waveguide through the evanescent tail of the propagating fundamental TE mode. To enable low-loss inter-layer coupling, we design a taper implemented in the α-Si layer. The 150 nm wide taper tip (*w*_tip_) ensures minimal back-reflections and compatibility with deep-UV lithography. By optimizing the taper geometry (taper length and final width), the undesired coupling into the higher-order modes can be avoided. The taper is gradually widened from 150 nm to a width *w*_α-Si_ of 1 µm over a length *l*_c_ of 60 µm. A side-view intensity profile of the inter-layer modal transfer is shown in Figure 3b, while mode profiles before (*x* = 0) and after (*x* = *l*_c_) the taper are plotted in Figure 3c. The transition efficiency between Si_3_N_4_ and α-Si layers is calculated to be 95% (insertion loss of −0.2 dB) at the 1.31 µm wavelength, according to our 3D FDTD simulations. Subsequently, the α-Si waveguide is tapered to a surface grating width (*w*_gc_) of 13.1 μm (Figure 1a). The width of the grating coupler in the *z*-direction is set to maximize the field overlap between the SMF-28 fiber mode and the dominant electric field component *E_z_*. The waveguide-to-grating taper length (*l*_t_) is 200 μm, yielding a transition efficiency of 99%.

## 3. Results and Discussion

### 3.1. Uniform Grating Coupler

**Figure 4:**
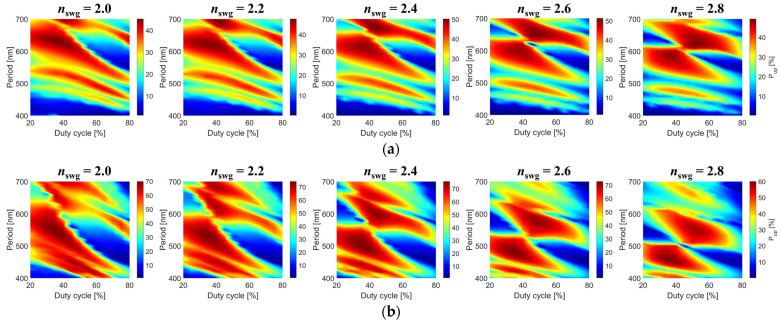

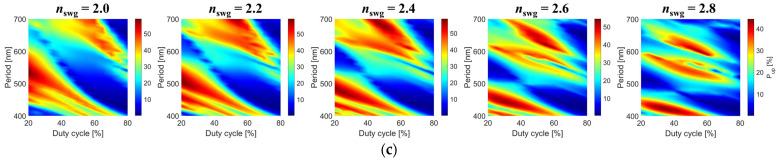
Two-dimensional upward radiation maps of uniform α-Si grating couplers with different SWG indices and α-Si thicknesses of (**a**) 110 nm, (**b**) 220 nm, and (**c**) 300 nm.

A parameter sweep with 2D FDTD simulations was conducted to optimize the performance of a uniform (non-apodized) grating coupler by mapping the directionality, field overlap, back-reflections, and coupling efficiency. We examined five structural parameters, namely the longitudinal (grating period, Λ, and duty cycle, *DC*), transversal (SWG metamaterial geometry), and vertical (*h*_b_ and *h*_α-Si_) dimensions. The grating period determines the lengths of the etched SWG (*l*_swg_) and non-etched (*l*_α-Si_) strips, where Λ = *l*_swg_ + *l*_α-Si_. The duty cycle is defined as the ratio between the non-etched α-Si block and the period, *DC* = *l*_α-Si_/Λ. For this analysis, a 50 nm thick oxide buffer is considered. The thickness of the α-Si grating layer is a design parameter aimed at maximizing the radiation towards the optical fiber above the chip. Figure 4 shows 2D design maps of the upward-radiated power as a function of the grating period and duty cycle. We considered α-Si thicknesses of 110 nm, 220 nm, and 300 nm and SWG metamaterial indices from 2.0 to 2.8.

The optimal uniform grating was found to have a period of 660 nm and a duty cycle of 40% with 220 nm thick α-Si. The associated lengths of the grating teeth and trenches are 264 nm and 396 nm, respectively. Comprising of 30 periods, the total length of the coupler is 19.8 μm. Since the grating is uniform, the radiated near-field exhibits an exponentially decaying profile, and the emission angle is 23° from the vertical axis. With air-filled trenches, the index contrast of the grating (~2) is too high. This results in a strong grating with a diffracted beam that is narrower than the 9.2 μm mode field diameter (MFD) of standard SMF-28 optical fibers at the 1.31 μm wavelength, yielding poor mode field overlap. Many surface couplers use controlled shallow etching [25] to form the grating with lowered coupling strength, and consequently, improved mode field overlap. This, however, brings an additional complexity to the fabrication process. Introducing a metamaterial with an equivalent refractive index of ~2.4 relaxes the grating strength while maintaining a single full-etch step fabrication process. As a result, the diffracted profile is widened and increases the grating–fiber field overlap to 80%. The equivalent metamaterial index of 2.4 is synthesized with an SWG period of Λ_SWG_ = 400 nm and a duty cycle of 67%. The grating radiates 71% of the waveguide power towards the fiber and 21% into the bottom Si substrate, which corresponds to a grating directionality of 77%.

**Figure 5:**
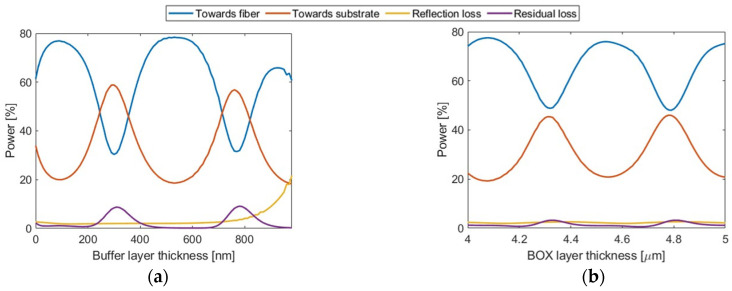
Grating radiation performance as a function of (**a**) buffer oxide thickness and (**b**) BOX thickness for the uniform coupler design.

The radiation performance of the α-Si grating is governed by interference arising from reflections at the interfaces of buffer oxide/Si_3_N_4_ and BOX/substrate. Figure 5a illustrates the radiation characteristics of the grating as a function of buffer oxide thickness. It shows that up to 50% of the upward radiated power is dependent on the thickness of the buffer oxide layer. Similarly, the dependence of the radiation performance on the BOX thickness is plotted in Figure 5b. The BOX layer’s influence on directionality is less pronounced, with a maxima-to-minima variation of approximately 25%. In contrast to the oxide buffer, which is a free design parameter in hybrid photonic platforms [16,54], variation in the BOX thickness is typically not an option in many photonic substrates. Therefore, the reduced dependence of the directionality on the BOX thickness is a significant advantage of our design.

**Figure 6:**
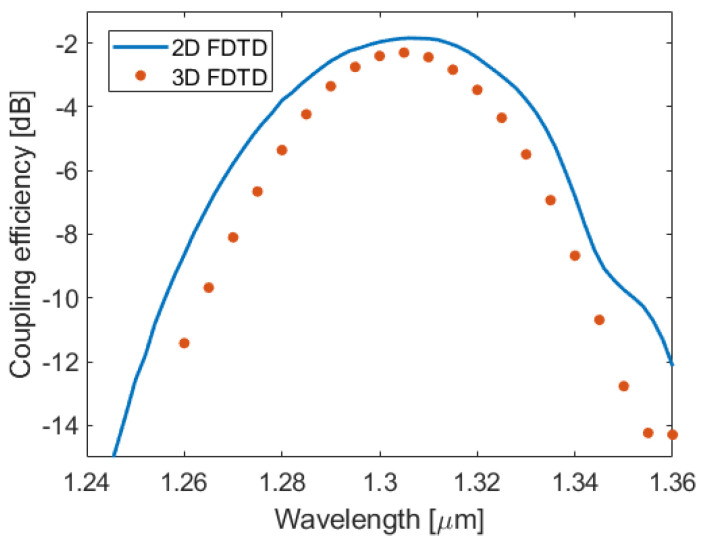
Coupling efficiency as a function of the wavelength for the uniform α-Si surface grating coupler.

The simulation results for the uniform grating design underscore the potential of this hybrid α-Si/Si_3_N_4_ platform. According to 3D FDTD simulations, a coupling efficiency of −2.3 dB is predicted for the uniform grating design, with back-reflections of 2.3% at the waveguide-to-grating junction. The coupling efficiency as a function of the wavelength is shown in Figure 6. The calculated 1 dB and 3 dB bandwidths are 30 nm and 50 nm, respectively. These findings mark a significant improvement over the performance achievable with SiN-only couplers [27]. Furthermore, the efficiency can be further enhanced by increasing the fiber–grating overlap through coupler apodization, as discussed in the following section.

### 3.2. Apodized Grating Coupler

**Figure 7:**
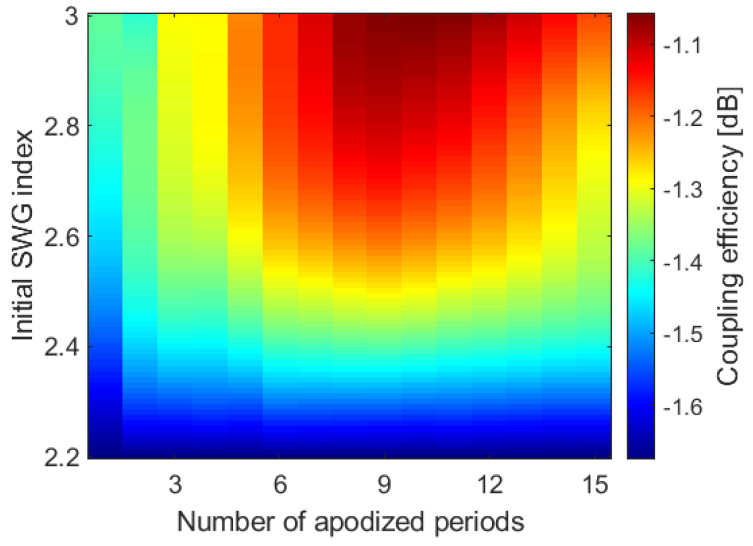
Apodization design map of the coupling efficiency as a function of the number of apodized periods and the initial SWG equivalent refractive index.

Further improvement of the coupling efficiency is achieved through SWG metamaterial engineering, which allows for a near-perfect mode match between the out-radiated grating field and the Gaussian-like optical fiber profile [26,36,42]. The apodization is performed by varying the refractive index of the SWG trenches along the direction of light propagation. This has the added benefit of lowering the effective index mismatch at the transition between the α-Si input waveguide and the surface grating, thus reducing back-reflections. Figure 7 shows a design map for the surface grating apodization obtained by evaluating the variation in coupling efficiency as a function of the number of apodized grating periods and the range of SWG metamaterial indices. The data were collected by conducting 2D FDTD simulations of the longitudinal cross-section of the device.

**Figure 8:**
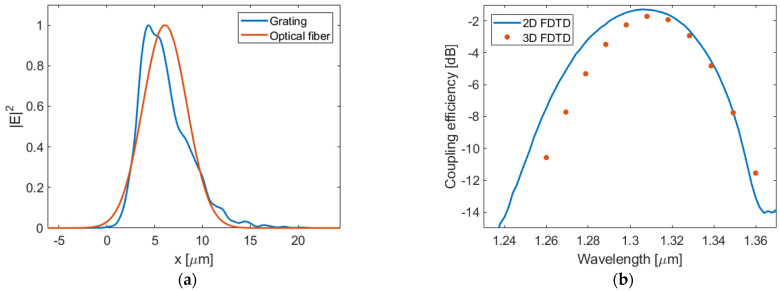
(**a**) Intensity profiles of the apodized diffracted beam and the near-Gaussian fiber mode. (**b**) Coupling efficiency as a function of the wavelength for the apodized grating coupler.

The optimized grating design has 7 periods in the apodized section followed by 23 periods with uniform coupling strength. The refractive index of the metamaterial trenches decreases from 3.0 to 2.2 in the apodized section, and the SWG period is 400 nm. The etched lateral gaps increase gradually from 28 nm to 163 nm. Although the calculated gap widths are limited by the SWG apodization profile [68,69], the utilization of ever-improving lithographic techniques with sub-50 nm feature size resolution, such as e-beam or immersion lithography [70], shows potential for faithful fabrication of many SWG-engineered devices in the future [64]. To keep a constant radiation angle, hence the linear phase profile of the out-radiated field, the grating period is chirped in a range from 590 nm to 691 nm over the full coupler length. The SWG-apodized α-Si surface grating has an improved fiber–grating field overlap of 96% and 92% according to 2D and 3D FDTD simulations, respectively. As a result, a total coupling efficiency of −1.7 dB is obtained at the nominal wavelength of 1.31 μm. The apodized grating design also yields a four-fold reduction in back-reflections, down to 0.8% (−21 dB). Figure 8a depicts a cross-section of the calculated intensity profiles of the SMF-28 fiber and the apodized grating field. The coupling efficiency as a function of the wavelength is plotted in Figure 8b. The optimized grating coupler provides 1 dB and 3 dB bandwidths of about 31 nm and 55 nm, respectively. Higher coupling efficiencies have been previously reported based on dual-layer configurations. However, they require thicker waveguide cores and metallic reflectors [54], or gratings etched into both the Si_3_N_4_ and Si layers, which complicate the fabrication process [53]. Our work represents an improvement of about 1.5 dB over standard grating couplers for Si_3_N_4_ photonics [27] while maintaining compatibility with a standard integration platform and a single-etch fabrication process.

## 4. Conclusions

In conclusion, we presented the design of an innovative grating coupler architecture aiming for Si_3_N_4_ photonic waveguides. By introducing a high-index α-Si overlayer, we circumvented the grating strength limitation inherent to the Si_3_N_4_ integration platform due to its moderate refractive index contrast. The use of SWG metamaterial apodization enhanced the coupling efficiency by 0.6 dB compared to the uniform grating, while maintaining a single-etch device fabrication without degrading the optical bandwidth. The calculated fiber–chip coupling efficiency was −1.7 dB at a wavelength of 1.31 µm with a 1 dB optical bandwidth of 31 nm. This work lays the foundation for developing high-efficiency grating couplers on the Si_3_N_4_ photonic platform, enabling opportunities for diverse applications, including high-speed data communication and on-chip quantum photonics.

## Data Availability

Data generated or analyzed in this study may be obtained from the contact author upon reasonable request.
